# A low-cost programmable pulse generator for physiology and behavior

**DOI:** 10.3389/fneng.2014.00043

**Published:** 2014-12-11

**Authors:** Joshua I. Sanders, Adam Kepecs

**Affiliations:** Neuroscience, Cold Spring Harbor Laboratory, Kepecs Lab, Cold Spring HarborNY, USA

**Keywords:** Pulse Pal, stimulator, open source, optogenetics, maple, arduino, pulse generator, timing

## Abstract

Precisely timed experimental manipulations of the brain and its sensory environment are often employed to reveal principles of brain function. While complex and reliable pulse trains for temporal stimulus control can be generated with commercial instruments, contemporary options remain expensive and proprietary. We have developed Pulse Pal, an open source device that allows users to create and trigger software-defined trains of voltage pulses with high temporal precision. Here we describe Pulse Pal’s circuitry and firmware, and characterize its precision and reliability. In addition, we supply online documentation with instructions for assembling, testing and installing Pulse Pal. While the device can be operated as a stand-alone instrument, we also provide application programming interfaces in several programming languages. As an inexpensive, flexible and open solution for temporal control, we anticipate that Pulse Pal will be used to address a wide range of instrumentation timing challenges in neuroscience research.

## Introduction

Patterned voltage pulse trains are commonly used in neuroscience research to precisely control stimulus isolators (Flaherty and Graybiel, 1994; Bisley et al., 2001; Cohen and Newsome, 2004; Histed et al., 2009), light sources for optogenetic manipulations (Boyden et al., 2005; Cardin et al., 2009), sensory stimuli (Soto-Faraco et al., 2002), and to synchronize events between instruments (Nikolic et al., 2009). Pulse trains can also be also triggered by a particular experimental contingency, providing closed-loop feedback at low latency (Girardeau et al., 2009; Venkatraman et al., 2009; Berényi et al., 2012; Newman et al., 2013). Laboratory instruments specialized for these purposes are commercially available, for example Master 8 (AMPI), PSG-2 (ISSI), Pulsemaster A300 (WPI), BPG-1 (Bak Electronics), StimPulse PGM (FHC Inc.) and Multistim 3800 (A-M Systems). Commercial solutions have been widely adopted, however their cost is a constraint in research and educational settings with limited funding. As proprietary instruments, researchers are also unable to add hardware or software features that would suit their unique needs: for instance, to implement a custom set of triggering rules in firmware, or to develop an interface to the device in a desired programming language. This flexibility can be especially beneficial for experimental design in systems neuroscience, where integration of custom instrumentation is frequently employed to measure and control behavior (Brunton et al., 2013), acquire neural data (Yamamoto and Wilson, 2008; Karlsson and Frank, 2009) and to stimulate the brain both electrically (O’Doherty et al., 2009) and optically (O’Connor et al., 2013).

**Figure 1:**
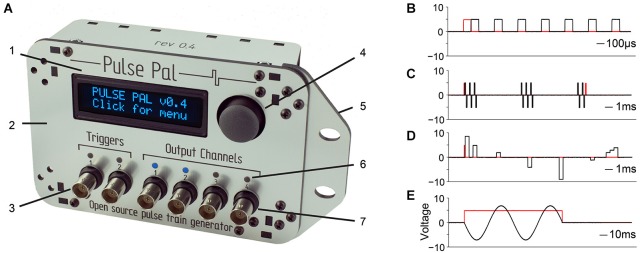
**Pulse Pal is a programmable pulse train generator. (A)** Pulse Pal front view, illustrating front panel features. 1: High contrast oLED screen permits programming with thumb joystick for stand-alone use. 2: Custom laser-cut acrylic enclosure. 3: Two optically isolated digital trigger channels. 4: Thumb joystick. 5: Rack-mount wing. 6: Channel activity indicators illuminate when channel voltage is not the set resting voltage (i.e., during a pulse). 7: Each of four analog output channels can be programmed with independent pulse trains and linked to either trigger channel.** (B–E)** Example pulse trains in black, acquired with an oscilloscope (see methods). Trigger voltage traces are shown in red. **(B)** A Pulse Pal output channel configured to deliver a train of 5 V, 100 µs square pulses with 200 µs intervals. **(C)** A train of biphasic +/−5 V 100 µs pulses, gated programmatically to produce pulse bursts. Trigger channel mode set to “toggle” aborts the ongoing pulse train in mid-burst when a second pulse arrives. **(D)** A train of 500 µs pulses with custom onset times and voltages. Pulses with consecutive onset times merge to form more complex waveforms (right). **(E)** A train of consecutive 100 µs pulses, whose voltages and onset times were configured to generate one period of a sine waveform. The output channel uses “loop mode”, to repeat the sine waveform until a parametrically specified pulse train end. The trigger channel mode was set to “pulse gated” mode, to abort the pulse train when its voltage returned low.

To address these needs, we developed Pulse Pal (Figure 1), an open source pulse train generator costing ~$210 (US) in easily obtained parts, with essential functionality comparable to commercial stimulators.

## System design

### Hardware

Pulse Pal was designed to be assembled at a laboratory soldering bench in approximately 1 h with minimal tools: a soldering iron, solder, a miniature Phillips head screw driver and a 4–40 tap. We provide instructions for ordering the necessary parts, assembling the device and programming firmware on the Pulse Pal wiki^1^. Hardware design files, drivers, firmware, and software interfaces to the device in MATLAB, Python and C++ are provided in a public repository.^2^ The assembled device and example pulse trains demonstrating key features are shown in Figure 1.

**Figure 2:**
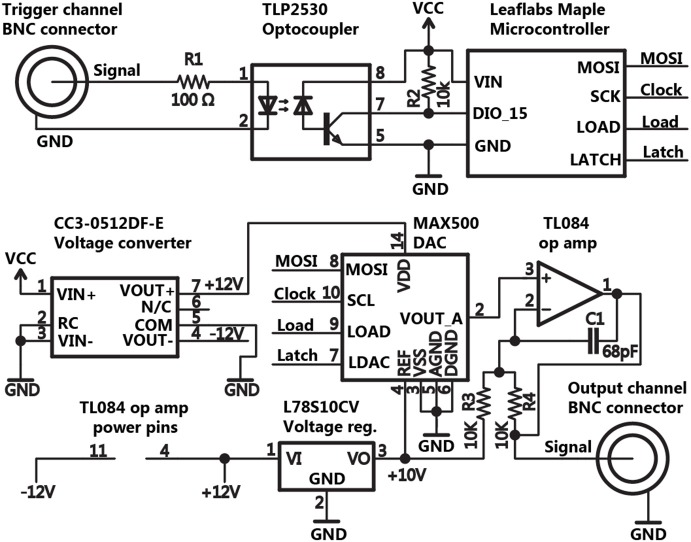
**Schematic of the basic circuit for triggering and pulse generation**. The schematic for Pulse Pal’s trigger and stimulation circuitry is shown for a single trigger and output channel, omitting duplicate circuitry for all other channels. Thumb joystick, oLED display, indicator LED and EEPROM connections with the microcontroller were omitted for clarity.

Pulse Pal’s essential triggering and stimulation circuit for a single trigger and analog output channel are shown in Figure 2. Pulse Pal passes incoming trigger logic signals through an optocoupler IC to protect microcontroller input pins and reduce potential for ground loops. Trigger signals are then read by Pulse Pal’s ARM Cortex M3 microcontroller (STM32F103RBT6, ST microsystems) provided as part of the open source Maple microcontroller platform (LeafLabs). The microcontroller generates analog waveforms by controlling an external 4-channel digital to analog converter (DAC) IC (MAX500ACPE+, Maxim Integrated Products), configured with bipolar output circuitry as specified in figure 9 of the MAX500 datasheet. This output circuitry consists of an Op Amp (TL084ACN, Texas Instruments) and two 10 k resistors (R3,R4) that divide the DAC reference voltage, collectively providing output voltages in the range of −10 to +10 V from each (otherwise unipolar) DAC output channel. A capacitor (C1) was added across each amplifier to suppress voltage transient overshoot. Voltage instructions are sent to the DAC over an 18 MHz hardware serial bus. For bipolar operation in the range of −10 to +10 V, the DAC requires power supplied at +/− 12 VDC. This supply is derived from Maple’s USB power supply with an integrated DC voltage converter (CC3-512DF-E, TDK Lambda). To set the range of the DAC to +/− 10 V, a separate 10 V reference voltage is provided to the DAC from the +12 V supply, using a linear voltage regulator (L78S10CV, ST Microsystems).

Further circuitry (not shown in Figure 2) was added to equip Pulse Pal for stand-alone operation. An oLED character display (NHD-0216KZW-AB5, Newhaven Display) and a two-axis pushbutton thumb joystick (802-30110A, P3 America) are used as an interface to program each channel’s parameters and test-trigger the device from a menu tree implemented in firmware. To retain parameters across power cycles, we added an external EEPROM IC (on a separate 9 MHz serial bus to accommodate the chip’s lower clock speed constraint; 25LC640A-I/P, Microchip Technology). An LED above each channel was added to indicate when the channel’s voltage is set to a value different from its programmed resting voltage (i.e., the channel is delivering a pulse). The complete schematic and circuit board layout are provided in the Pulse Pal repository, as files for Eagle printed circuit board (PCB) software (CadSoft) and as GERBER files for PCB manufacture.

### Software

The ARM processor that subserves Pulse Pal was programmed with custom firmware, written in the LeafLabs derivative of the Arduino language—a C++ based programming language for AVR and ARM microcontrollers. Pulse Pal’s firmware was programmed to execute its main loop every 50 µs when delivering pulse trains. Loop execution is triggered by a hardware timer, provided as an internal feature of the microcontroller. On each loop cycle, the microcontroller updates the DAC, reads trigger-channel logic and any single-byte USB serial instructions, computes the logic of the current cycle’s voltage transitions, and adjusts indicator LED status. USB serial instructions employ a system of single byte op-codes, enabling a software client to program and trigger Pulse Pal, abort ongoing stimulation, set fixed voltages for output channels, or set logic values of Maple’s I/O lines for debugging. To distinguish it from subsequent updates, the firmware version used to acquire performance data for the present publication is provided in a dedicated folder in the code repository.

**Figure 3:**
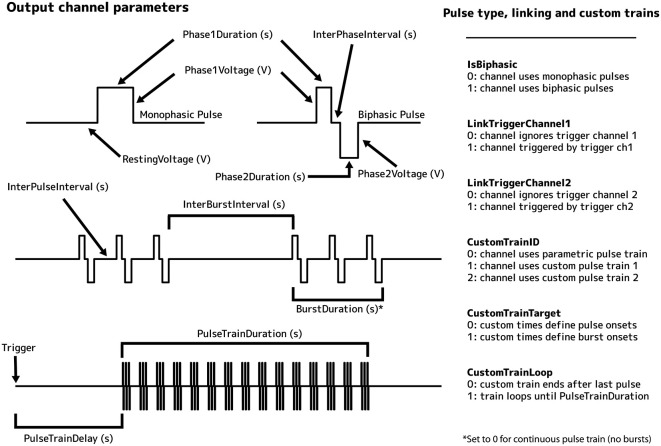
**Illustration of output channel parameters**.

Pulse Pal is programmed either using its thumb joystick interface or via USB by setting channel parameters (indicated below by their cross-platform syntax in italic, and illustrated in Figure 3 for output channels). A single parameter for each trigger channel, *TriggerMode*, controls how it interprets incoming logic. Three trigger modes are provided: “normal”, “toggle” and “pulse gated”. In normal mode, an incoming logic pulse triggers all linked output channels, but subsequent triggers are ignored during playback. In toggle mode, subsequent triggers terminate ongoing pulse trains on linked output channels. In pulse gated mode, pulse trains are triggered by a low to high logic transition on the trigger channel, and terminated by the subsequent high to low transition if it occurs during playback.

The stimulus parameters of each output channel can be independently programmed. Output channels can deliver either parametric pulse trains or custom pulse trains, for which each pulse has a specified onset time and voltage. Pulse shape and frequency are defined by 7 parameters: *IsBiphasic* (0 if pulses are monophasic, 1 if biphasic), *Phase1Voltage* (voltage of the first phase, −10 V to +10 V), *Phase2Voltage* (same range), *Phase1Duration* (duration of the first phase, 0.1 s to 3600 s), *InterPhaseInterval* (the time between phases of a biphasic pulse), *Phase2Duration*, and *InterPulseInterval* (time between pulses). Pulse trains are defined by an additional 4 parameters: *BurstDuration* (time during which the underlying pulse train is gated “on”) *BurstInterval* (a period alternating with *BurstDuration* during which the pulse train is gated “off”), *PulseTrainDelay* (time between trigger and pulse train onset), and *PulseTrainDuration* (duration of the pulse train). Two custom pulse trains of up to 1,000 pulses each can be defined, where the user specifies the onset time and voltage of each pulse. Use of a custom train on an output channel is selected by setting the channel’s *CustomTrainID* parameter to a non-zero value (1 or 2, specifying which train). For custom trains, two additional parameters are configurable: *CustomTrainTarget* (for onset times and voltages; 0 if these refer to pulses, 1 if these refer to bursts of pulses), and *CustomTrainLoop* (0 if pulse train ends after final pulse defined, 1 if pulse train loops from trigger until the value of *PulseTrainDuration*). Each output channel has three additional settings: *LinkTriggerChannel1*, *LinkTriggerChannel2* and *RestingVoltage*. The first two of these parameters specify which trigger channels control the output channel. The third specifies the output channel’s resting voltage between pulse phases, pulses and pulse trains (0 V by default).

Design files for a device enclosure that can be laser cut from a single sheet of 30.48 × 30.48 cm (12″ × 12″) acrylic are provided in the repository. Raster-engraved text in the design indicates channel and USB port identities. Light pipes (PLP2, Bivar) press into holes above each channel, routing light from indicator LEDs on the circuit board to the enclosure surface. The enclosure attaches to the circuit board with screws fastened to threaded circuit board stand-offs, and contains a removable wing for attaching the device to a server rack (shown in Figure 1A).

## Measures of reliability and precision

To validate Pulse Pal as a practical solution for stimulus control, we tested the precision and reliability of the shortest pulses the device can process on both trigger and output channels, and several other properties relevant for neurophysiology research. All tests were performed on a single Pulse Pal device, connected to a controlling computer (Macbook Pro, Apple).

**Figure 4:**
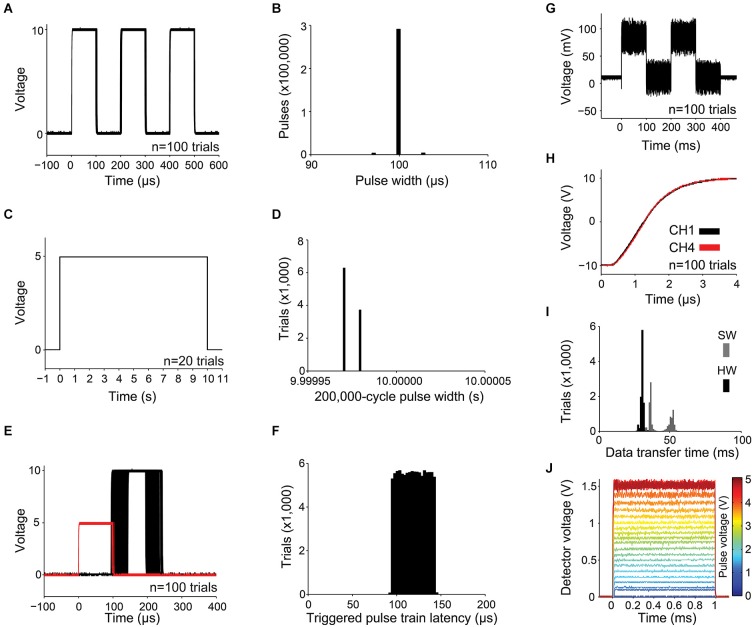
**Measurements of precision and reliability. (A–B)** For a train of three 100 µs pulses with 100 µs pulse intervals: **(A)** the first 100 waveforms captured with the oscilloscope are shown superimposed, and **(B)** distribution of pulse widths measured from 100,000 3-pulse trains, captured as in **(A)**. **(C–D)** For a train of a single 10 s pulse: **(C)** waveforms from the first 20 trials and **(D)** 10,000 pulse widths. **(E)** The latency of a pulse train of one 10 V, 100 µs pulse captured from an output channel (shown in black for 100 trials) was measured with respect to a 5 V, 100 µs pulse delivered to a linked trigger channel (shown in red). **(F)** Distribution of pulse train latencies for 100,000 trials. **(G)** 100 superimposed 78.1 mV pulses, showing the smallest possible increment of the digital to analog converter and channel noise caused by digital feed-through from the SPI bus. **(H)** Simultaneous and rapid settling of the voltage on channels 1 and 4 when delivering a +10 V pulse from a resting voltage of −10 V. **(I)** USB transfer time is shown for a 5,006 byte message containing pulse times and voltages for a 1,000-pulse custom train. Transfer time was measured with hardware (HW, black; using firmware modified to indicate transmission start and end with a voltage pulse) and software (SW, gray; using the controlling computer’s clock). **(J)** 1 ms pulses of light, produced by controlling a blue diode laser with Pulse Pal, converted to voltage with an Si transimpedence amplified photodetector (PDA10A, ThorLabs), and captured with an oscilloscope. Single traces are shown for voltage pulses ranging from 78 mV to 5 V in amplitude.

While output channels are updated once per execution of the microcontroller’s 50 µs main loop, the shortest configurable pulse is restricted to 100 µs (to ensure that the smallest output channel pulse can also reliably trigger the device). To measure the precision of a 100 µs pulse, we programmed Pulse Pal to deliver a train of three 100 µs pulses, separated by 100 µs intervals on all 4 output channels each time a software trigger was detected. Pulse Pal’s first output channel was connected to a digital oscilloscope (DS1102D, Rigol). Pulse Pal was then software-triggered 100,000 times over 24 h by a custom test script written in MATLAB r2013a (Mathworks) on the controlling computer. After each trigger event, the resulting waveform was returned from the oscilloscope to the computer. Pulse Pal generated a unique waveform of three pulses after each trigger, demonstrating high software trigger reliability. The first 100 pulse trains are shown superimposed in Figure 4A, aligned to first pulse onset to demonstrate pulse jitter. The pulse widths of all 300,000 pulses are shown in Figure 4B. Cycle widths ranged from 96.9 µs to 102.9 µs, and 99.97% of pulses were within 3 µs of 100 µs.

Next, we measured clock drift, to ensure that the timing variability we observed in 100 µs-long pulses did not propagate. We programmed Pulse Pal to deliver a single 10 s pulse (spanning 200,000 microcontroller loop cycles) when triggered. We captured the resulting waveform on 10,000 trials with an analog capture device (NI USB-6210, National Instruments), sampled at 100 kHz. 100 example pulse waveforms are shown in Figure 4C, and all 10,000 pulse widths are shown in Figure 4D. All pulses measured either 9.99998 s or 9.99997 s, corresponding to a consistent clock drift of 3 µs/s with respect to the NI USB-6210 clock.

To measure pulse train latency and trigger channel reliability, we connected two Pulse Pals in series. The first was soft-triggered by the computer on each of 100,000 trials. It generated a single 5 V, 100 µs square pulse simultaneously on two output channels—one delivered to the first trigger channel of the second Pulse Pal, and the other to an oscilloscope (see Figure 4H for a separate experiment demonstrating the simultaneity of these pulses). The second Pulse Pal generated a single 100 µs pulse on each output channel when its first trigger channel was triggered, which was captured from one output channel by a separate oscilloscope channel. In Figure 4E, 100 trials are shown. Trigger pulses from the first (triggering) Pulse Pal are shown in red, and pulses from the second Pulse Pal in black. All 100,000 pulses captured were unique waveforms, indicating high hardware trigger reliability. Output channel latency for all 100,000 trials ranged relatively uniformly between 91.0 and 146.7 µs (Figure 4F).

Next we sought to determine whether channel noise was low enough that a single bit DAC increment produced a non-overlapping change in voltage. Since the MAX500ACPE+ DAC that drives Pulse Pal’s output channels has 8-bits of precision mapped across a 20 V range (−10 V to +10 V), its least significant bit (LSB) increments the channel voltage by 78.1 mV. Therefore we set up PulsePal to trigger two 78.1 mV pulses 100 times (shown in Figure 4G). Fluctuations in voltage on individual trials ranged as much as 8 mV about the mean in the 100 ms interval prior to pulse train onset, and as much as 65 mV about the mean during the first 100 ms pulse. The increased noise during playback was mostly attributable to digital feed-through from the SPI channels controlling the DAC (data not shown), but remained significantly less than the DAC’s minimum voltage increment. While several board layout and circuit modifications could be implemented by researchers wishing to further reduce digital feed-through (for instance, by optical isolation of the SPI bus), Pulse Pal can exploit the full bit-width of its DAC in its present form, rendering it useful for many control applications in neuroscience instrumentation. The resting voltage of the output channel was programmatically set to 0 V, but was measured in this experiment to be 10.55 mV (within the 15 mV “zero code error” specified for the MAX500ACPE+ DAC in its datasheet), indicating that an offset from the 0 V set-point was present but slight.

In experiments with precisely timed events, it is useful to produce signals that occur simultaneously. Therefore we measured the simultaneity of output channel updates by comparing pulses triggered on the first and last output channels. We set the first and fourth output channels to a resting voltage of −10 V, delivered 100 +10 V pulses, and captured the rising waveform of each pulse with the oscilloscope (Figure 4H). On all trials, the output voltage on both channels settled within 100 mV of +10 V after 3.5 µs. This measurement also confirmed that DAC and output amplifier slew rates were fast enough to produce 100 us pulses useful for most applications in neuroscience research.

In many experiments, pulse train parameters and timing data must be updated rapidly in response to recently acquired information. Since the same microcontroller controls pulse timing and USB communication, Pulse Pal cannot be updated while a pulse train is being delivered. Therefore we sought to ensure that updates could be achieved rapidly between experimental trials. We measured the USB data transfer speed by sending a 1000-pulse train (5006 bytes) 100 times, from the Pulse Pal MATLAB client to Pulse Pal. For a measure of the hardware transfer speed without client-side software overhead, Pulse Pal’s firmware was modified to indicate the start of data transfer by setting output channel 1–5 V, and the end of data transfer by returning channel 1–0 V. The resulting pulse was captured by the oscilloscope on each trial. The client-side transfer time was separately measured for the blocking MATLAB serial fwrite command, by flanking it with tic and toc commands. Transfers completed in 26–35 ms (averaging 171 KB/s), while client-side overhead cost an additional 12 ms on average (Figure 4I). Consistent with this transfer speed measurement, a separate transfer updating *all* of Pulse Pal’s channel parameters for all channels (163 bytes) completed on the hardware side in less than 1 ms (data not shown).

Finally, we sought to verify Pulse Pal’s suitability for precise optical control in optogenetic experiments, by using it to control a 447 nm diode laser’s timing and intensity. We connected the laser (BML447-50FLD, Lasermate Group) through an optical fiber (M31L02, ThorLabs) to a silicon transimpedence amplified photodetector (PDA10A, ThorLabs), delivered 1 ms pulses from a Pulse Pal output channel to the laser power supply’s analog input, and captured the resulting waveforms with an oscilloscope (DS1102D, Rigol). Pulses ranged in amplitude from 78 mV to 5.0 V in 78 mV increments. In Figure 4J, the single traces captured for each voltage are shown superimposed, skipping every second voltage for clarity. Pulse Pal elicited precisely gated pulses of light from the laser, with programmatic (albeit slightly nonlinear) control of light intensity.

## Applications

### Light timing and intensity control for optogenetics

Pulse Pal was originally developed in a laboratory setting, to provide an intuitive and affordable way to achieve fine temporal control in optogenetics experiments (Pi et al., 2013). In these studies, Pulse Pal was used to control a laser coupled to an optical fiber as in Figure 4J, providing precisely timed pulse trains to photostimulate specific classes of interneurons. In this role, Pulse Pal provides a simple and open alternative to commercial pulse train generators (e.g., Master 8 (AMPI), PSG-2 (ISSI), Pulsemaster A300 (WPI), BPG-1 (Bak Electronics), StimPulse PGM (FHC Inc.) and Multistim 3800 (A-M Systems).

### Sensory pattern generation with low latency gating

In the same research study, Pulse Pal doubled as a programmable waveform generator, providing simple, low-latency acoustic stimuli for a Go/No-Go perceptual decision task. In this application, each output channel directly drove a separate amplified speaker. Beyond the simple cues used in these experiments, temporally patterned auditory and visual pulse stimuli are often used to study the algorithmic basis for human and animal decision making. A binaural Poisson click stream (Sanders and Kepecs, 2012; Brunton et al., 2013) can be generated using Pulse Pal’s custom pulse trains, where a 100 us, 1 V pulse delivered to an amplified headphone speaker generates a precisely timed audible click. For visual stimuli, each channel can be configured to produce precisely timed visual flashes (Zylberberg et al., 2012), by gating a commercial LED driver (e.g., BuckPuck, LED Dynamics). Thus, the stimuli can be triggered and stopped with much lower latency and higher temporal precision than a commercial sound card or computer video display (Kleiner et al., 2007). For sensory decision making experiments that require fine temporal control, Pulse Pal provides a simple and open alternative to custom instrumentation.

### General analog control of laboratory instruments

Several neuroscience instruments use analog signals as an interface to control device parameters. Some examples are galvanometer mirrors for laser scanning stimulation (e.g., GVSM002, Thor Labs), and monochromators to measure spectral tuning in optogenetics (e.g., Polychrome V, Till Photonics). Programmatic control of unipolar voltage can be accomplished inexpensively in some cases with microcontroller platforms (e.g., Arduino) or low-cost automation devices (e.g., U3, LabJack). However, many devices (including the two listed above) require control voltages in the industry standard range of −10 V to +10 V, necessitating expensive, proprietary computer hardware (e.g., NI PCIe-6323, National Instruments). For these applications, Pulse Pal provides an inexpensive way to achieve analog control.

### Closed-loop feedback in electrophysiology

As a temporal control tool, Pulse Pal complements a growing array of open source *acquisition* tools for neuroscience research, which have become available in recent years. These range from electrophysiology acquisition systems (Rolston et al., 2009; Voigts et al., 2013a) to electrode interface devices (Voigts et al., 2013b) and data acquisition software tools (Brainard, 1997; Pologruto et al., 2003; Englitz et al., 2013; Campagnola et al., 2014). Pulse Pal has been formally integrated into the software for one of these tools, the Open Ephys electrophysiology acquisition system,^3^ where it is provided as one method for low latency closed loop feedback.

## General discussion

For our research, we required a pulse generator with high precision at timescales relevant for alignment of stimulation events to action potentials (pulse time jitter at least a factor of 10 less than an action potential width; Figures 4A,B), low clock drift (Figures 4C,D), first pulse latency (Figures 4E,F) comparable to short mammalian action potentials (Kandel et al., 2000) and high reliability (100% of 300,000 soft triggers, 100% of 100,000 100 µs trigger pulses). In developing Pulse Pal, we recognized that simplified control of voltage pulse timing is a general need, and expanded the project’s scope to meet five additional design objectives: low material cost ($210 USD), stand-alone functionality (Figure 1), bench-side assembly with common tools (see illustrated guide on wiki), support for common computing platforms and programming languages (WinXP, Win7, OSX, Ubuntu 14.04; MATLAB, C++, Python) and comprehensive online documentation.

In fulfilling these objectives, Pulse Pal provides a general resource for precise temporal control of stimulation and environmental cues in the laboratory. It encapsulates the problem of temporal pattern generation for many applications in physiology and psychophysics, where in lieu of commercial instrumentation, this control problem had often been addressed *ad-hoc* by writing custom software for microcontrollers (da Silva Pinto et al., 2011; Weick et al., 2011; Bugaj et al., 2013; Haikala et al., 2013; Ohayon et al., 2013; Smear et al., 2013; Inagaki et al., 2014; Klapoetke et al., 2014).

Pulse Pal’s parametric approach to stimulation features trigger logic rules and stimulus pattern motifs commonly implemented in neuroscience research, however custom applications may require the device to perform less common functions. Unlike its commercial counterparts, Pulse Pal’s firmware is provided in the public domain with an open source license. To facilitate access, the firmware was written in the Arduino language^4^ —a reduced set of C++ syntax with extensive online documentation for developers who lack a programming background. The adaptation of Arduino for Pulse Pal’s microcontroller platform^5^ exposes further functionality specific to the ARM Cortex M3 microcontroller. We anticipate that this lower barrier to entry will be exploited by researchers using Pulse Pal’s hardware, firmware and software as a starting point for tailored applications beyond its present niche.

## Conflict of interest statement

The authors declare that the research was conducted in the absence of any commercial or financial relationships that could be construed as a potential conflict of interest.

## Footnotes

^1^https://sites.google.com/site/pulsepalwiki/home

^2^https://github.com/PulsePal/PulsePal

^3^www.open-ephys.org

^4^www.arduino.cc

^5^www.leaflabs.com
